# Risk of second malignancies in patients with early-stage classical Hodgkin's lymphoma treated in a modern era

**DOI:** 10.1002/cam4.405

**Published:** 2015-01-26

**Authors:** Melissa H LeMieux, Abhishek A Solanki, Usama Mahmood, Steven J Chmura, Matthew Koshy

**Affiliations:** 1 Department of Radiation and Cellular Oncology, University of ChicagoChicago, Illinois; 2 Division of Radiation Oncology, University of Texas M.D. Anderson Cancer CenterHouston, Texas; 3 Department of Radiation Oncology, University of Illinois HospitalChicago, Illinois

**Keywords:** Early-stage classical Hodgkin's lymphoma, radiotherapy, second malignancy, SEER

## Abstract

Second malignancies remain an issue affecting morbidity and mortality in long-term survivors of early stage Hodgkin's lymphoma (HL). We undertook this study to determine if treatment in the modern era resulted in decreased second malignancies. Patients diagnosed with stage I–II cHL between 1988 and 2009 who received radiation therapy (RT) were selected from the Surveillance, Epidemiology, and End Results (SEER) database. Freedom from second malignancy (FFSM) was estimated using the Kaplan–Meier method. Univariate analysis (UVA) was performed using the Log-Rank test, and included age, gender, year of diagnosis, and stage. Multivariable analysis (MVA) was performed using Cox Proportional Hazards modeling. The study cohort included 8807 patients. The median age at diagnosis was 32 years (range: 2–85). The majority of patients had stage II disease (*n* = 6044, 69%), 597 (7%) had extranodal involvement (ENI), and 1925 (22%) had B symptoms. Median follow-up for the entire cohort was 7.2 years (range: 0–22). Five hundred twenty-three (6%) patients developed a second malignancy. Median latency to second malignancy was 5.8 years (range: 0.1–21.5). Of the 523 patients that developed a second malignancy, 228 (44%) occurred in the first 5 years, 139 (27%) were diagnosed between years 5–10, and 156 (30%) beyond 10 years. The 10 year FFSM for patients treated between 1988 and 1999 was 93.0% versus 95.1% for patients treated between 2000 and 2009 (*P* = 0.04), On MVA, treatment between 2000 and 2009 was associated with a HR for second malignancy of 0.77 (95% Confidence Interval: 0.62–0.96, *P* = 0.02) compared to the treatment between 1988 and 1999. Our analysis suggests that in patients treated with RT for stage I or II cHL, treatment prior to 2000 had a slightly higher risk of second malignancy compared to treatment in 2000 and later. Further studies, with longer follow-up of patients treated in the modern era are needed to confirm these findings.

## Introduction

Historically, radiation therapy (RT) alone was used as definitive treatment for early-stage (stage I and II) classical Hodgkin's lymphoma (cHL) using an extended field (EF) RT technique (treating the clinically involved and adjacent uninvolved sites) 1. Later, the addition of chemotherapy to RT regimens allowed for improved disease outcomes 2,3. However, the observation of late treatment-related side effects provided motivation to de-escalate treatment, especially for these early-stage patients 4. In the late 1990s and early 2000s, there was a shift in the paradigm of combined-modality therapy (CMT) from using larger, extensive RT fields to more limited, involved fields (treating only the clinically involved lymph node regions) while providing similar disease outcomes 2,5,6.

One of the main toxicity concerns with RT is the possibility that patients may develop a radiation-related second malignancy. Retrospective data suggest that larger radiation fields are associated with an increased risk of second malignancies and a higher integral radiation dose is associated with an increased risk of death from a second malignancy 7–9. The incidence of breast and lung cancers, among others, has been documented to be elevated in cHL patients after treatment, resulting in poor outcomes 10–17. However, a recently published SEER analysis of early-stage cHL has demonstrated that the use of RT in these patients improves overall survival (OS) without increasing the rate of second malignancies 18.

This study aimed to determine if treatment in a more modern era was associated with improved outcomes compared to an older treatment era.

## Methods and Materials

### Patients

The patient cohort was selected from the Surveillance, Epidemiology, and End Results (SEER) database, which includes 28% of the U.S. population and data from 18 cancer registries (SEER-18). It contains patient data such as primary tumor site, age at diagnosis, gender, histologic type, stage of disease at diagnosis, first course of treatment, follow-up, and cause of death.

Patients diagnosed with cHL between 1988 and 2009 were selected. Patients were included only if they received RT as part of their initial treatment. Patients whose extent of disease corresponded to the current American Joint Committee on Cancer stage I and II were included; those with stage III or IV or an unknown stage were excluded. Patients were excluded if they had a previous or simultaneous hematologic malignancy. Patients with any other type of cancer prior to their cHL diagnosis were included. The last potential date of follow-up in our cohort was December 31, 2009. Institutional Review Board exemption for this study was obtained from the University of Chicago Institutional Review Board.

Patient characteristics examined included year of diagnosis, stage, age at diagnosis, gender, primary disease site, extranodal involvement (ENI), and the presence of B symptoms. Information regarding the details of RT (field size, beam energy, dose, and fractionation) and the use of chemotherapy was not available from the SEER database. For all analyses, patients were divided into two groups by year of diagnosis (1988–1999 and 2000–2009). This grouping was chosen as the year 2000 was when portions of the preliminary data from the H8-U trial was presented, showing equivalent outcomes of CMT with either IF-RT or EF-RT (subtotal nodal radiation).

Freedom from second malignancy (FFSM) was the primary endpoint of this analysis. Time to second malignancy (latency) was defined as the interval from diagnosis of cHL to the date of diagnosis of the second malignancy. FFSM was defined as the percentage of patients without a second malignancy. OS was defined as the interval from the date of diagnosis to the date of death from any cause. Cause-specific survival (CSS) was defined as the interval from cHL diagnosis to the date of death from cHL. Patients who died of causes other than cHL were censored for the CSS.

### Statistical analysis

FFSM was estimated using the Kaplan–Meier method. Univariate analysis (UVA) was performed for FFSM and included age, gender, year of diagnosis, and stage. The Log-rank test was used for comparisons between categorical groups and the trend test was used for continuous variables (age of diagnosis). Multivariable analysis (MVA) was performed using Cox Proportional Hazards modeling and included all covariates used in UVA. All statistical tests were two-sided and a *P* value of 0.05 was considered statistically significant. The data were analyzed using Stata/MP version 12.1 (Statacorp, College Station, TX).

## Results

### Study population

We identified 8807 patients with stage I or II cHL who received RT as part of their treatment. The median follow-up for the entire cohort was 7.2 years (range: 0–22). The median follow-up for the 1988–1999 cohort was 13.7 years (range: 0.3–21.9) and 4.8 years (range: 0–9.9) for the 2000–2009 cohort. The median age at diagnosis for the entire cohort was 32 (range: 2–85) years. Four thousand five hundred thirty-eight patients were female (52%). The most common involved lymph nodes sites were as follows: multiple regions (*n* = 4339, 49%); head and neck (*n* = 2351, 27%); and intrathoracic (*n* = 902, 10%). Most patients had stage II disease (*n* = 6044, 69%), 597 (7%) had ENI, and 1925 (22%) had B symptoms. Table1 describes additional patient and tumor characteristics by year cohort.

**Table 1:** Patient and tumor characteristics.

Characteristic (total population, *n* = 8807)	1988–1999 (*n* = 3463)	2000–2009 (*n* = 5344)
Median follow-up (months)	164 (range: 3–263)	57 (range: 0–119)
Median age (years)	31 (range: 2–85)	32 (range: 3–85)
Age (years)		
<16	183 (5%)	358 (7%)
16–25	943 (27%)	1366 (26%)
26–35	1008 (29%)	1391 (26%)
36–45	588 (17%)	966 (18%)
46–55	301 (7%)	539 (10%)
56–65	191 (6%)	301 (6%)
≥66	249 (7%)	423 (8%)
Gender		
Male	1648 (48%)	2621 (49%)
Female	1815 (52%)	2723 (51%)
Stage		
I	1310 (38%)	1453 (27%)
II	2153 (62%)	3891 (73%)
Primary site		
Lymph nodes of multiple regions	1658 (48%)	2681 (50%)
Lymph nodes of head, face, and neck	1048 (30%)	1303 (24%)
Intrathoracic lymph nodes	344 (10%)	558 (10%)
Lymph nodes of axilla or arm	161 (5%)	221 (4%)
Lymph nodes of inguinal region or leg	91 (3%)	126 (2%)
Lymph nodes, NOS	68 (2%)	230 (4%)
Other sites	93 (3%)	225 (4%)
Extranodal involvement		
No	3264 (94%)	4946 (93%)
Yes	199 (6%)	398 (7%)
B symptoms		
No	2832 (82%)	4050 (76%)
Yes	631 (18%)	1294 (24%)

NOS, not otherwise specified.

### Second malignancies

During the study's follow-up period, 523 (6%) of patients developed a second malignancy. The median latency to second malignancy was 5.8 years (range: 0.1–21.5). Two hundred twenty-eight (44%) patients developed a second malignancy in the first 5 years after their cHL diagnosis, 139 (27%) between years 5–10, and 156 (30%) of the second malignancies were diagnosed greater than 10 years after their index diagnosis. Overall, the 10 year FFSM was 93.6%.

### Univariate and multivariate analysis

**Figure 1:**
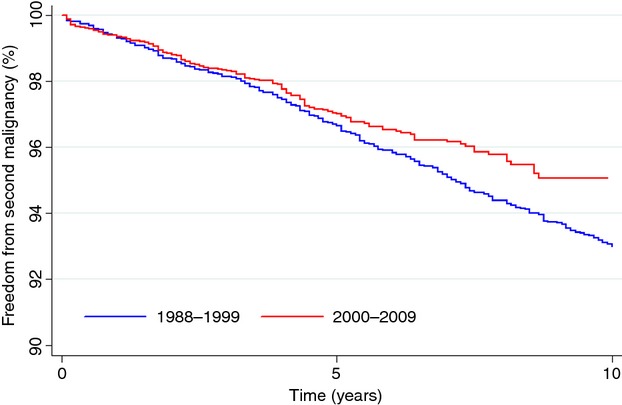
Freedom from second malignancy (FFSM). FFSM in patients diagnosed in 1988–1999 versus 2000–2009.

UVA revealed treatment in 2000 or later (*P* = 0.04), younger age at diagnosis (*P* < 0.001), and stage II (*P* < 0.01) were associated with improved FFSM (Table2). The 5 year FFSM for patients treated from 1988 to 1999 was 96.6% versus 97.1% for patients treated from 2000 to 2009, and 93.0% versus 95.1% at 10 years (*P* = 0.04) (Figure1). On multivariate analysis, treatment before 2000 (*P* = 0.02) and older age (36 years and older) (*P* < 0.01) was associated with a worse FFSM, with a trend toward worse FFSM for females (*P* = 0.08) (Table3).

**Table 2:** Univariate analysis for freedom from second malignancy.

Covariate	5 year	10 year	*P*-value
Year of diagnosis			
1988–1999	96.6%	93.0%	0.04
2000–2009^1^	97.1%	95.1%	
Age at diagnosis			
<16	99.2%	98.3%	<0.01^2^
16–25	99.1%	97.2%	<0.01
26–35	98.7%	97.6%	
36–45	96.9%	93.4%	
46–55	95.2%	88.0%	
56–65	87.3%	74.2%	
≥66	86.2%	73.4%	
Gender			
Male	96.6%	93.3%	0.39
Female	97.1%	93.9%	
Stage			
I	95.3%	90.6%	<0.01
II	97.6%	95.1%	

1 The last patient in this group is censored at 119 months.

2 Trend test.

**Table 3:** Multivariate analysis for developing a second malignancy.

Covariate	Hazard ratio (95% confidence interval)	*P*-value
Age (years)		
<16	(Referent)	0.12
16–25	1.78 (0.86–3.70)	0.07
26–35	1.96 (0.95–4.04)	<0.01
36–45	4.72 (2.30–9.68)	<0.01
46–55	7.39 (3.56–15.35)	<0.01
56–65	18.86 (9.15–38.86)	<0.01
≥66	18.04 (8.69–37.47)	
Year of diagnosis		
1988–1999	(Referent)	0.02
2000–2009	0.77 (0.62–0.96)	
Gender		
Male	(Referent)	0.08
Female	1.17 (0.98–1.39)	
Stage		
I	(Referent)	0.32
II	0.91 (0.76–1.09)	

The 5 year OS for the 1988–1999 and 2000–2009 diagnosis groups was 90.2% versus 92.1%, respectively, while the 10 year OS was 83.2% versus 87.4% (*P* < 0.01). The CSS for the 1988–1999 and 2000–2009 diagnosis groups at 5 years was 95.3% versus 96.0%, and 94.0% versus 95.0% at 10 years (*P* = 0.06), respectively.

### Second malignancy sites

In the entire cohort, the most common locations of secondary malignancy were breast (18%), lung (15%), prostate (8%), skin (8%), and bone marrow (7%) (Table4).

**Table 4:** Second malignancy characteristics by year-group of diagnosis.

	1988–1999 (*n* = 3463)	2000–2009 (*n* = 5344)
Second malignancies (*n*)	376 (10.9%)	147 (2.8%)
Secondary tumor location (selected sites)	*n* (%)	*n* (%)
Breast	77 (21%)	15 (10%)
Lung	61 (16%)	17 (12%)
Prostate	26 (7%)	17 (12%)

## Discussion

Classical HL is a disease with excellent long-term cure rates 5,6,19. Over time, treatment has been modified to try to decrease long-term toxicities while preserving the favorable outcomes. Patients with cHL are known to have an increased risk of developing second malignancies and the concern for development of RT-induced second malignancy has been the reason physicians have attempted to decrease the size of the RT treatment field over time 5,7,8,19.

This study evaluates the outcomes of patients with stage I-II cHL who received RT as part of their treatment regimen from a national cancer registry and found that diagnosis in a more modern era (2000–2009) was associated with an improved FFSM when compared to patients diagnosed in an older era (1988–1999) (95.1% vs. 93.0%, *P* = 0.04). This difference persisted even after controlling for age, gender, and stage.

Our analysis demonstrated that older age (36 years and older) was associated with a worse FFSM. This is likely due to the increased risk of any second malignancy with increasing age (both radiation-related and non-radiation-related) when compared to the risk of second malignancies in younger people.

There was also a trend toward worse FFSM for women. When examining the locations of the second tumors by year of diagnosis, the 2000–2009 cohort demonstrated a lower proportion of breast cancers, when compared to those treated before 2000. This is not surprising given that the group treated in the more modern era likely had less breast tissue in the treatment field when compared to those treated in the older era 20. Previous studies have noted breast and lung cancers to be elevated in treated cHL patients 10–17. Franklin et al. performed a meta-analysis of the second malignancy rate after IF-RT versus EF-RT and found more breast cancers in the EF-RT patients when compared to the IF-RT patients (OR=3.25, *P* = 0.04), but no difference in lung cancers rates (*P* = 0.22) 21. This study also found a higher proportion of second breast cancers and all second malignancies in patients treated in the older era, but no difference in lung cancers. There an increase in the proportion of prostate cancer in the modern era, which is likely due to increased screening. However, caution must be used when interpreting these results, given the shorter follow-up time in the 2000–2009 cohort. This could affect both the time to develop a second cancer (latency) and the age at diagnosis of second malignancy (important with age-related cancers).

The 10 year CSS for both groups were similar (94.0% and 95.0%, *P* = 0.06), while the 10 year OS for 2000–2009 group was improved when compared to the 1988–1999 group, 83.2% versus 87.4%, respectively (*P* < 0.01). This difference in survival could be due a combination of shorter follow-up time in these patients and less toxic treatment in the modern group. In addition to decreasing the risk of second cancers, use of smaller radiation fields has other potential benefits such as decreased dose to the heart and subsequent cardiac toxicity.

This study has the inherent limitations of a retrospective review. One important limitation is the lack of detail of RT. Specifically, the information regarding the RT specifics of field size, dose, fractionation, or beam energy is not available. While there is not a specific point in time that can be identified when all physicians transitioned from using EF-RT versus IF-RT for cHL patients, we used the 1999 versus 2000 cut point to approximate this transition. It is also unknown whether the second cancer was in the field of the prior radiation. Information on the type of chemotherapy the patients may have received is also unavailable, which can affect rates of second cancers and disease outcomes. Whether patients had unfavorable disease features, which would affect treatment and survival, is also unknown. Additionally, conclusions regarding the incidence of second malignancies cannot be made at time points later than the duration of follow-up in our cohort, with more second malignancies likely to be seen with longer follow-up. The median follow-up of the 1988–1999 cohort was 13.7 years (range 0.3–21.9 years) and 4.8 years (range 0–9.9 years) in the 2000–2009 group. We were able to detect a significant difference after a relatively short follow-up period in the 2000–2009 group. However, because the majority of second malignancies occur after 10 years or more after treatment, these results must be interpreted with caution. Further studies will be needed to determine if the difference we see after 4.8 years of follow-up in the modern cohort persist with longer follow-up.

In conclusion, our study demonstrates that patients with early stage Hodgkin's disease treated in a more modern era were associated with a slightly lower incidence of developing second malignancies, while achieving similar survival outcomes.
